# Loss of HIF-1β in macrophages attenuates AhR/ARNT-mediated tumorigenesis in a PAH-driven tumor model

**DOI:** 10.18632/oncotarget.8297

**Published:** 2016-03-23

**Authors:** Nina Henke, Nerea Ferreirós, Gerd Geisslinger, Martina G. Ding, Silke Essler, Dominik C. Fuhrmann, Theresa Geis, Dmitry Namgaladze, Nathalie Dehne, Bernhard Brüne

**Affiliations:** ^1^ Institute of Biochemistry I, Goethe-University Frankfurt, 60590 Frankfurt, Germany; ^2^ Institute of Clinical Pharmacology, *Pharmazentrum Frankfurt*, Goethe-University Frankfurt, 60590 Frankfurt, Germany

**Keywords:** xenobiotics, CYP1A1, DNA damage, breast cancer, fibrosarcoma

## Abstract

Activation of hypoxia-inducible factor (HIF) and macrophage infiltration of solid tumors independently promote tumor progression. As little is known how myeloid HIF affects tumor development, we injected the polycyclic aromatic hydrocarbon (PAH) and procarcinogen 3-methylcholanthrene (MCA; 100 μg/100 μl) subcutaneously into myeloid-specific *Hif-1α* and *Hif-2α* knockout mice (C57BL/6J) to induce fibrosarcomas (n = 16). Deletion of *Hif-1α* but not *Hif-2α* in macrophages diminished tumor outgrowth in the MCA-model. While analysis of the tumor initiation phase showed comparable inflammation after MCA-injection, metabolism of MCA was impaired in the absence of *Hif-1α*. An *ex vivo* macrophage/fibroblast coculture recapitulated reduced DNA damage after MCA-stimulation in fibroblasts of cocultures with *Hif-1α^LysM−/−^* macrophages compared to wild type macrophages. A loss of myeloid *Hif-1α* decreased RNA levels of arylhydrocarbon receptor (AhR)/arylhydrocarbon receptor nuclear translocator (ARNT) targets such as *Cyp1a1* because of reduced Arnt but unchanged *Ahr* expression. Cocultures using *Hif-1α^LysM−/−^* macrophages stimulated with the carcinogen 7,12-dimethylbenz[a]anthracene (DMBA; 2 μg/ml) also attenuated a DNA damage response in fibroblasts, while the DNA damage-inducing metabolite DMBA-trans-3,4-dihydrodiol remained effective in the absence of *Hif-1α*. In chemical-induced carcinogenesis, HIF-1α in macrophages maintains ARNT expression to facilitate PAH-biotransformation. This implies a metabolic activation of PAHs in stromal cells, i.e. myeloid-derived cells, to be crucial for tumor initiation.

## INTRODUCTION

Polycyclic aromatic hydrocarbons (PAHs), like 3-methylcholanthrene (MCA), are procarcinogenic components of air pollutants, tobacco smoke, or overcooked food [1]. According to the National Cancer Institute up to 30% of human cancers in the US are attributed to tobacco smoke and inhaled pollutants (www.cancer.gov). In mouse models, carcinogens like PAHs induce tumors dose-dependently. During early phases of PAH-induced tumor development immune reactions are compromised [2]. Carcinogenic activation of PAHs involves their biotransformation by cytochrome P450 (CYPs) to more reactive diol-epoxides, which bind DNA and thereby induce DNA damage [3]. Among CYP-isoforms, only CYP1A1, CYP1A2, and CYP1B1 activate PAH and its PAH-dihydrodiol. The expression of CYP1A2 is restricted to the liver, while CYP1A1 and CYP1B1 are also expressed in extra hepatic tissue such as lung and mammary glands [4]. PAHs activate the arylhydrocarbon receptor (AhR) and provoke dimerization with the arylhydrocarbon receptor nuclear translocator (ARNT), which causes transcriptional induction of CYP enzymes [3]. So far, the metabolic activation of PAHs has mainly been analyzed in hepatocytes [3, 5-7], while little is known on the involvement of macrophages in this process [8]. However, a recent publication elucidated the importance of Langerhans cells, the skin tissue-resident macrophages, for metabolic activation of PAHs [9]. Besides the metabolism of environmental carcinogens, AhR is also activated by endogenously formed ligands, referring to the development of immune tolerance and increased survival in cancer [10, 11]. In contrast to the application of chemical carcinogens, other mouse models have been established to study tumor formation based on genetic alterations. A common model for breast cancer are transgenic mice expressing the polyoma virus middle T oncoprotein (PyMT) within breast epithelial cells. In this model tumor formation is spontaneous and occurs in all transformed mice [12].

Immune cells like macrophages significantly contribute to all phases of cancer development, a process known as cancer immunoediting [13, 14]. Chronic inflammation, fostered by immune competent macrophages, drives cancer formation for example following infections with *human papillomaviruses* or *Helicobacter pylori* [14-16]. If tumor elimination fails, tumors start to expand but still engage interactions with immune cells to perpetuate tumor-associated inflammation [17]. Macrophages are fundamental in supporting cancer immunoediting at the initial phase of tumorigenesis [18]. Moreover, signals in the tumor microenvironment polarize macrophages to a pro-tumor or alternatively-activated phenotype, to promote malignant conversion and immune escape [18]. These tumor-associated macrophages (TAMs) frequently accumulate in hypoxic areas of the tumor [19].

Hypoxia develops due to inadequate blood supply or enhanced oxygen consumption of proliferating cells. As a consequence, hypoxia-inducible factor (HIF) is activated in tumors. HIF is a heterodimer composed of an oxygen-sensitive HIF-α (HIF-1α, HIF-2α, or HIF-3α) subunit and constitutively expressed HIF-1β, which is also known as ARNT [19, 20]. Under normoxic conditions, the HIF-α subunit is hydroxylated by prolyl hydroxylases (PHDs). Following hydroxylation, the α-subunits are recognized by the von Hippel-Lindau (VHL) protein, causing their poly-ubiquitination and proteasomal degradation. Under hypoxia PHDs are inhibited, hydroxylation of HIF-α is reduced, thus allowing its accumulation and dimerization with ARNT in the nucleus [19]. Interestingly, HIF-α shares its dimerization partner HIF-1β/ARNT with AhR. So far, the interaction between the HIF- and the AhR-pathway has been discussed controversially [5, 21, 22].

DNA binding of HIF upregulates a variety of target genes promoting angiogenesis, metastasis, and proliferation [23]. High levels of HIF in tumors correlate with a poor clinical patient outcome [23, 24]. HIF-inhibitors are under consideration as anticancer agents, although the influence of HIF in stromal cells on tumor progression is poorly understood [24-27]. An improvement of conventional chemotherapy for example was shown by targeting PHD2, and thereby activating HIF in endothelial cells, enhancing tumor perfusion and drug delivery [28]. Only few studies addressed the impact of HIF in macrophages on tumor progression, indicating tumor-promoting properties of both HIF-isoforms, although mechanisms largely remain uncertain [19, 29, 30].

To elucidate the role of HIF in macrophages during carcinogenesis, we analyzed tumor formation in wild type (wt) mice or mice carrying a myeloid-specific knockout of *Hif-1α*, *Hif-2α*, and *Hif-1α/2α* in the transgenic PyMT breast cancer model as well as chemical carcinogenesis, induced by subcutaneous injection of MCA. Most strikingly, mice lacking *Hif-1α* in macrophages did not develop tumors in the MCA model. Mechanistically, a knockout of *Hif-1α* decreased CYP-mediated metabolic MCA-activation in macrophages and a DNA damage response in fibroblasts.

## RESULTS

### Myeloid-specific deletion of HIF-1α attenuates tumor formation in MCA-induced carcinogenesis

**Figure 1:**
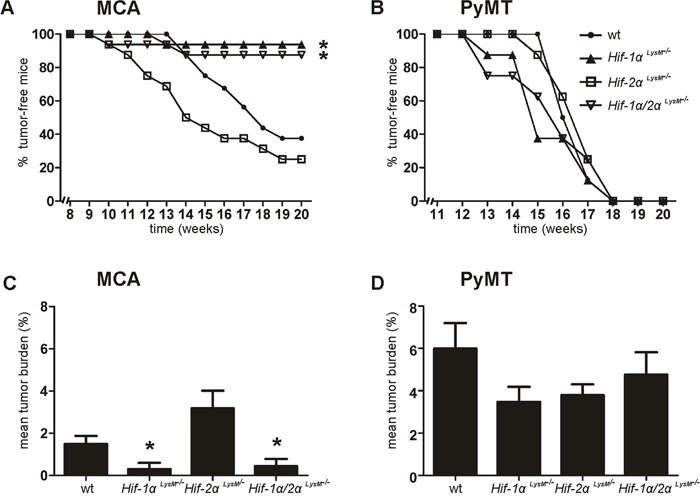
Role of HIF in tumorigenesis **A.** Development of 3-methylcholanthrene (MCA)-induced fibrosarcomas (n = 16 per genotype) and **B.** development of polyoma virus middle T oncoprotein (PyMT) breast cancer (n = 8 per genotype) in wt C57BL/6J, myeloid *Hif-1α^LysM−/−^*, *Hif-2α^LysM−/−^* and *Hif-1α/2α^LysM−/−^* mice was assessed by palpation twice weekly. **C.** Mean tumor burden [(tumor weight/body weight)/number of mice)] in MCA-injected and **D.** PyMT mice was calculated at the end of the experiments. Bar graphs show mean values ± SEM. * P < 0.05 compared to wt mice.

To explore the role of HIF-α isoforms in carcinogenesis, wt and myeloid conditional knockout mice lacking *Hif-1α* and/or *Hif-2α* were used in two different tumor models. *Hif-1α* expression in peritoneal macrophages (PMs) was reduced by 87% in *Hif-1α^LysM−/−^* macrophages compared to wt mice (Supplementary Figure 1A). *Hif-2α* was expressed in wt macrophages and reduced by 97% in *Hif-2α^LysM−/−^* cells (Supplementary Figure 1B). To validate functional consequences of the knockout, we analyzed HIF target gene expression under hypoxia. Adrenomedullin (*Adm*), a classical HIF-1 target gene, was reduced in macrophages isolated from *Hif-1α^LysM−/−^* mice or double knockout macrophages, but not in macrophages from *Hif-2α^LysM−/−^* mice (Supplementary Figure 1C). In contrast, Arginase1 (*Arg1*), a preferred HIF-2 target gene, was downregulated in *Hif-2α^LysM−/−^* and double knockout macrophages (Supplementary Figure 1D). To analyze tumor formation, we used two different tumor models in C57BL/6J wt, *Hif-1α^LysM−/−^*, *Hif-2α^LysM−/−^*, and *Hif-1α/2α^LysM−/−^* mice. First, MCA was injected subcutaneously into the right flank of mice to induce fibrosarcomas at the site of injection. Compared to the chemical-induced cancer, tumor formation was also followed in the transgenic PyMT breast cancer model by crossing C57BL/6J conditional *Hif-1α^LysM−/−^*, *Hif-2α^LysM−/−^*, and *Hif-1α/2α^LysM−/−^* mice with mice expressing the PyMT oncoprotein. In the MCA-model 63% of wt mice displayed a tumor after 20 weeks (Figure 1A). *Hif-2α^LysM−/−^* mice showed a more rapid tumor formation but the overall rate of tumor development was not significantly different from wt mice. In contrast, tumor frequency in *Hif-1α^LysM−/−^* as well as in *Hif-1α/2α^LysM−/−^* mice was significantly diminished, basically being absent. Depicted in Figure 1C, the tumor burden in mice lacking *Hif-1α* (mean tumor burden: 0.303% ± 0.303) and *Hif-1α/2α* (0.438% ± 0.347) was smaller than in wt mice (1.496% ± 0.379), whereas the mean tumor burden in *Hif-2α^LysM−/−^* mice (3.185% ± 0.833) was not significantly enhanced compared to wt mice. Tumors of *Hif-2α^LysM−/−^* and wt mice were analyzed for immune cell infiltration and vascularization, but no differences comparing wt with *Hif-2α^LysM−/−^* mice became detectable (Supplementary Figure 2A-2D). In the PyMT breast cancer model, no statistical differences in tumor development occurred (Figure 1B) and tumor burden was marginally but not significantly affected (Figure 1D). These data identify HIF-1α as an important factor in tumor formation in chemical PAH-induced carcinogenesis.

### Loss of HIF-1α in macrophages impairs metabolic activation of MCA *in vivo*

**Figure 2:**
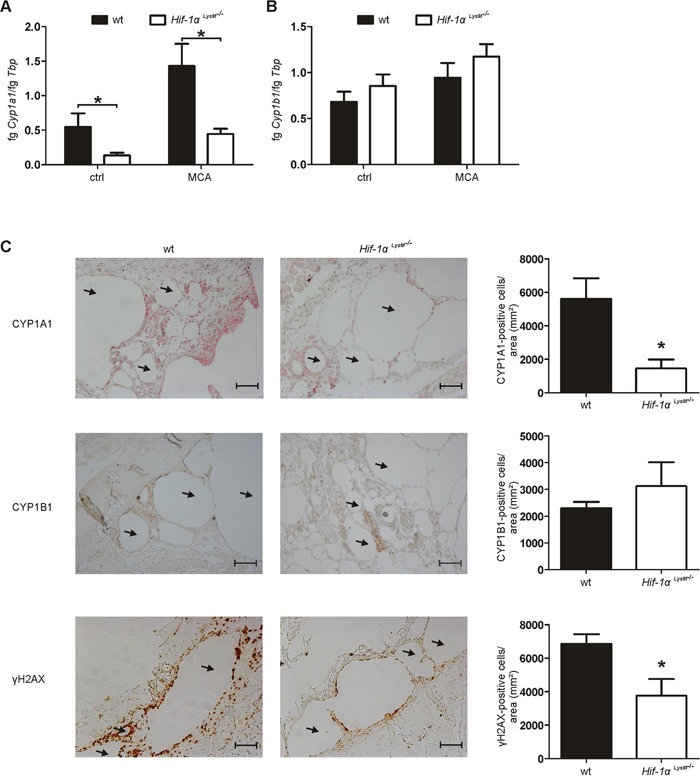
*Cyp* expression and DNA damage in *Hif-1α^LysM−/−^* mice **A.** *Cyp1a1* and **B.** *Cyp1b1* mRNA expression in whole skin tissue in close proximity to MCA-injection sites 5 days after application was assessed by qPCR. mRNA levels were normalized to *Tbp*. Values give the mean ± SEM of n = 7. * P < 0.05. **C.** Representative images and quantification of skin sections 5 days after MCA-injection, stained for CYP1A1 (red), CYP1B1 (brown) and γH2AX (brown) by immunohistochemistry. Arrows indicate lipid droplets. Scale bars: 100 μm. Values are the mean ± SEM of n = 3 (*HIF-1α^LysM−/−^)/* 4 (wt), * P < 0.05 compared to wt.

The reduced/absent tumor incidence in *Hif-1α^LysM−/−^* mice suggests differences in tumor initiation as a likely mechanism for reduced tumor outgrowth. To characterize the role of HIF-1α in myeloid cells at early stages of carcinogenesis, tissue samples from the site of MCA-as well as corn oil (control)-injections were obtained after 5 days. This time point was chosen to allow immune cell infiltration [31] and provided similar results to the analysis performed at day 10 (data not shown). As MCA was dissolved in corn oil, lipid droplets with encapsulated MCA formed in the skin in close proximity to injection sites. The formation and occurrence of these droplets were similar in skin tissue of wt and *Hif-1α^LysM−/−^* mice (data not shown). Five days after MCA-injection mainly neutrophils and fewer macrophages invaded the tissue surrounding lipid droplets. Skin of wt and *Hif-1α^LysM−/−^* mice showed a similar pattern of neutrophil and macrophage accumulation after the application of MCA, whereas minor neutrophil infiltration was detected following control injections, i.e. corn oil (Supplementary Figure 3A). Furthermore, the production of inflammatory cytokines such as interleukin (IL)-1ß or tumor necrosis factor α (TNFα), reported to be involved in MCA-induced tumor formation [31, 32], revealed no differences comparing wt and *Hif-1α^LysM−/−^* mice (Supplementary Figure 3B, 3C). Thus, differences in inflammation at sites of tumor initiation appear highly unlikely to explain a reduced tumor outcome in the absence of myeloid HIF-1. Therefore, metabolic activation of the MCA pre-carcinogen was analyzed 5 days after MCA-injection. In general, the MCA-metabolizing enzyme CYP1A1 was expressed in macrophages in the skin as shown by immunohistochemistry (Supplementary Figure 4A). The expression of *Cyp1a1* mRNA in *Hif-1α^LysM−/−^* mice was diminished by approximately 75% under control conditions at sites of corn oil-injection compared with wt mice (Figure 2A). Reduced *Cyp1a1* expression became more pronounced following MCA injection, whereas the expression of *Cyp1b1* was similar in both genotypes in skin tissue (Figure 2B). These findings were confirmed by immunohistochemistry, detecting CYP1A1 and CYP1B1 in skin around lipid droplets. Consistent with mRNA expression, CYP1A1 signals were diminished around lipid droplets in skin of *Hif-1α^LysM−/−^* mice compared with wt mice (Figure 2C), while CYP1B1 expression around the injection site remained unchanged (Figure 2C). We then used a functional read-out of metabolic activation and analyzed DNA damage by immunohistochemistry staining for the phosphorylated form of the histone H2AX (γH2AX). Interestingly, DNA damage was detected in tissue close to the MCA-containing lipid droplets of wt mice, but was largely reduced in skin of *Hif-1α^LysM−/−^* mice (Figure 2C). We could not detect differences in DNA damage in the skin of HIF-2*α^LysM−/−^* compared to wt mice (Supplementary Figure 4B). These results suggest that the loss of *Hif-1α*, but not of *Hif-2α*, diminishes CYP1A1-mediated metabolic activation of MCA in skin *in vivo*, thereby reducing DNA damage.

### HIF-1α-deficient macrophages reveal an altered expression of xenobiotic metabolizing enzymes and limit DNA damage *ex vivo*

**Figure 3:**
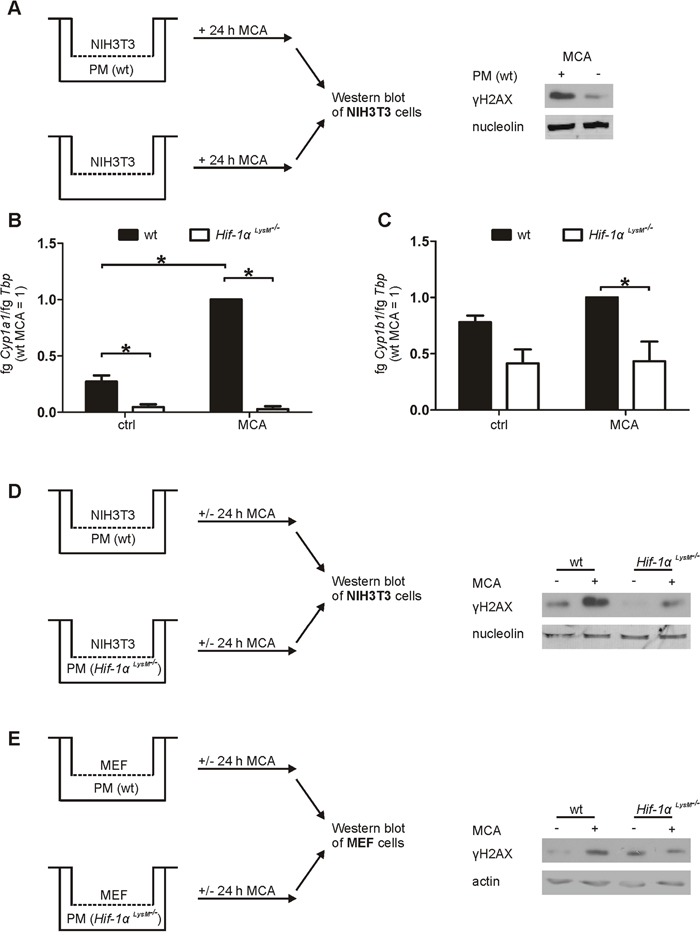
Impaired *Cyp1a1* and *Cyp1b1* expression in *Hif-1α^LysM−/−^* macrophages and fibroblast DNA damage in an *in vitro* coculture **A.** Schematic illustration of the NIH3T3/peritoneal macrophages (PMs) coculture set up, stimulated with MCA for 24 h. A representative Western blot of phosphorylated γH2AX in NIH3T3 cells cocultured with or without PMs is shown. Nucleolin served as a loading control. **B.** *Cyp1a1* and **C.** *Cyp1b1* mRNA expression of PMs that were isolated from wt and *Hif-1α^LysM−/−^* mice and stimulated with MCA or dimethylsulfoxide (DMSO: ctrl) for 8 h assessed by qPCR. mRNA levels were normalized to *Tbp*. The ratio of *Cyp1a1* or *Cyp1b1* and *Tbp* in wt mice after stimulation with MCA was set to 1. Mean values ± SEM of n = 4 are presented (each n contains 3 mice/genotype). * P < 0.05. **D.** Illustration of the cocultures of NIH3T3 cells with PMs isolated from wt or *Hif-1α^LysM−/−^* mice **E.** and the cocultures of MEF cells with PMs isolated from wt or *Hif-1α^LysM−/−^* mice, stimulated with MCA or DMSO for 24 h. Representative Western analysis of phosphorylated H2AX in NIH3T3 is shown. Nucleolin or actin served as loading controls.

To verify molecular mechanisms of reduced metabolic MCA-activation, biotransformation was analyzed *ex vivo*. As it was recently published that tissue-resident macrophages, i.e. Langerhans cells, are the key player for metabolic activation of PAHs in the skin, we used macrophages in our *ex vivo* studies to explore the role of HIF-1α in metabolic activation of MCA and subsequent DNA damage [9]. *In vivo*, transformation of fibroblasts induces fibrosarcomas after MCA-application [33]. Therefore, we established a coculture model of PMs and NIH3T3 fibroblasts or mouse embryonic fibroblasts (MEF) to measure DNA damage *ex vivo*. Although stimulation with MCA caused some DNA damage in NIH3T3 cells in the absence of macrophages, DNA damage was substantially increased in NIH3T3 cells cocultured with wt macrophages without affecting viability or proliferation within the 24 h incubation period (Figure 3A, Supplementary Figure 5A, 5F-5I). PMs isolated from *Hif-1α^LysM−/−^* and wt mice were then analyzed for mRNA expression of the MCA-metabolizing enzymes *Cyp1a1* and *Cyp1b1*. Stimulation with MCA induced the expression of *Cyp1a1* (Figure 3B), while the increase in *Cyp1b1* was marginal (Figure 3C). Similar to the results in skin, the expression of *Cyp1a1* was drastically reduced by about 97% in PMs of *Hif-1α^LysM−/−^* mice compared to wt PMs under control conditions (Figure 3B). Also, a massive reduction of *Cyp1a1* mRNA was detected after MCA-application in macrophages lacking *Hif-1α*. In addition, PMs of *Hif-1α^LysM−/−^* mice also reduced *Cyp1b1* expression by roughly 47% under control conditions as well as after MCA-stimulation (Figure 3C). To assess functional consequences of reduced *Cyp1a1* and *Cyp1b1* mRNA expression, we cocultured NIH3T3 fibroblasts or MEFs with wt PMs or with PMs isolated from *Hif-1α^LysM−/−^* mice. Consistent with our observations *in vivo*, H2AX phosphorylation, a marker of DNA damage, was lower in NIH3T3 cells and MEFs that were cocultured with PMs of *Hif-1α^LysM−/−^* mice, but not with PMs of *Hif-2α^LysM−/−^* mice, compared to NIH3T3 cells or MEFs cocultured with wt PMs (Figure 3D, 3E, Supplementary Figure 5B-5E). Apparently, macrophages are important to induce DNA damage in fibroblasts after MCA-application requiring CYP expression and subsequent metabolic PAH-activation in myeloid cells.

### CYPs account for metabolic activation of MCA

**Figure 4:**
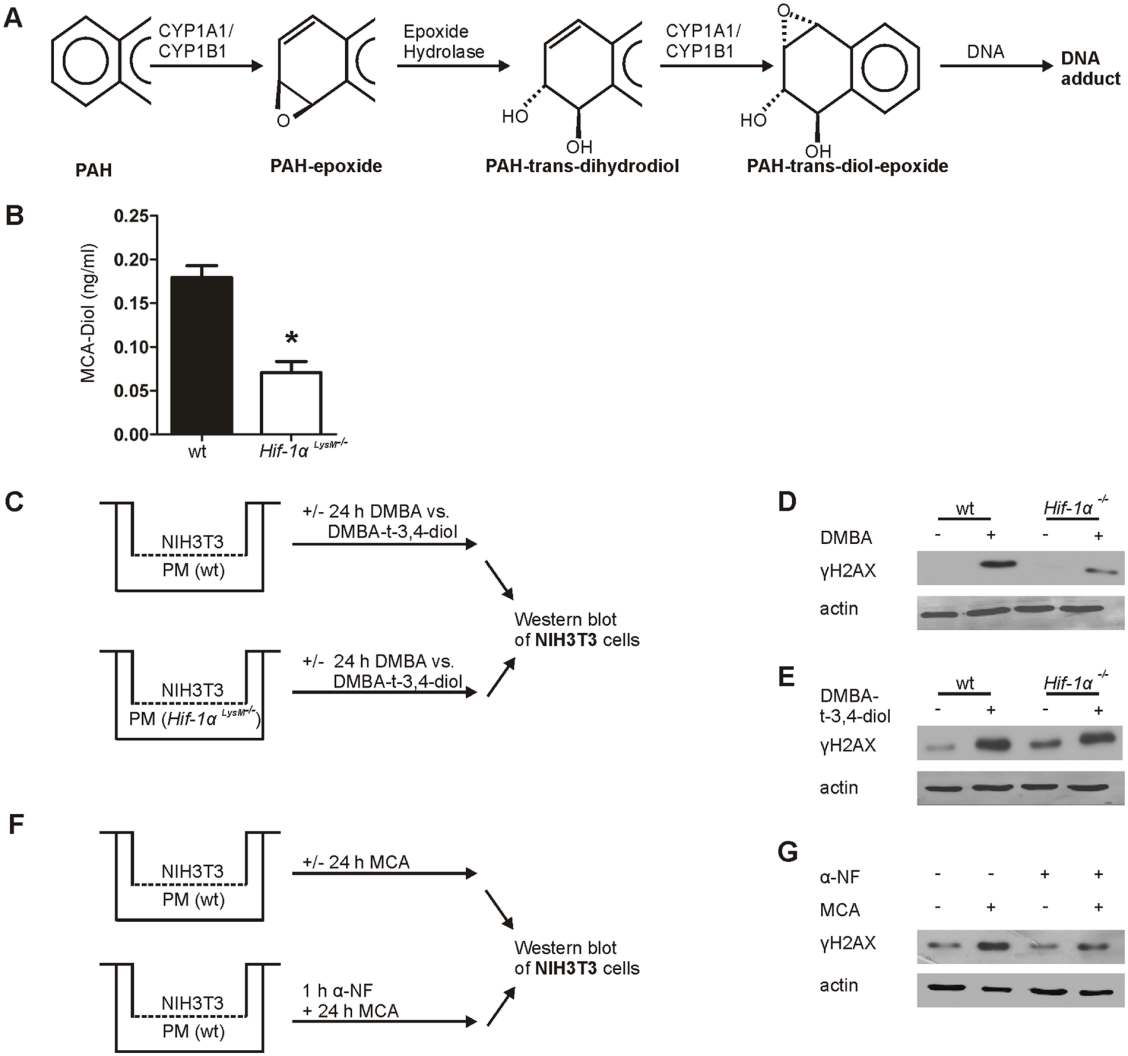
Metabolism of 3-methylcholanthrene (MCA) in peritoneal macrophages **A.** Schematic overview of the metabolic activation of polycyclic arylhydrocarbons (PAHs). **B.** Quantification of MCA-Diol in the supernatants of isolated peritoneal macrophages (PMs) from wt and *Hif-1α^LysM−/−^* mice stimulated with MCA for 8 h and quantified by LC-MS/MS. Bars are the mean ± SEM of n = 4 (each n containing 3 mice/genotype). * P < 0.05 compared to wt. **C.** Schematic illustration of the coculture of NIH3T3 cells with PMs isolated from wt or *Hif-1α^LysM−/−^* mice stimulated with 7,12-dimethylbenz[a]anthracene (DMBA), DMBA-trans-3,4-dihydrodiol (DMBA-t-3,4-diol), or DMSO for 24 h. Representative Western blot of phosphorylated γH2AX in NIH3T3 cell stimulated with **D.** DMBA or **E.** DMBA-t-3,4-diol from 4 experiments is shown. Actin served as a loading control. **F.** Schematic illustration of the coculture of NIH3T3 and wt PMs, prestimulated for 1 h with or without 10 nM α-napthoflavone (α-NF), a CYP-inhibitor, following stimulation with MCA or without for 24 h. **G.** Representative Western blot of phosphorylated γH2AX in NIH3T3 cells cocultured with wt peritoneal macrophages from 5 experiments is shown. Actin served as a loading control.

To determine the potential of PMs isolated from *Hif-1α^LysM−/−^* mice, showing a reduced *Cyp1a1* and *Cyp1b1* expression, to activate MCA, we quantified the metabolite MCA-trans-9,10-dihydrodiol (MCA-Diol) by LC-MS/MS. CYP1A1 and CYP1B1 hydroxylate PAHs like MCA to a MCA-9,10-epoxide (PAH-epoxide; Figure 4A), which subsequently is converted by epoxide hydroxylase-1 (*Ephx-1*) to MCA-9,10-transdihydrodiol that is again hydroxylated by CYP1A1 or CYP1B1 to a more reactive MCA-9,10-trans-diol-epoxide. Therefore, PMs of wt and *Hif-1α^LysM−/−^* mice were stimulated with MCA for 8 hours and supernatants were collected for LC-MS/MS measurements. MCA-Diol formation was significantly reduced in supernatants of *Hif-1α^LysM−/−^* PMs compared to wt PMs (Figure 4B). In cells that were stimulated with DMSO only (ctrl) no metabolites were detected in the supernatants (data not shown) and the expression of *Ephx-1*, which catalyzes the formation of MCA-dihydrodiol from MCA-epoxide, did not differ between wt and *Hif-1α* knockout-cells (Supplementary Figure 6A).

To prove the relevance of PAH-metabolism in myeloid cells in causing DNA damage we used the PAH 7,12-dimethylbenz[a]anthracene (DMBA) and its commercially available active metabolite DMBA-trans-3,4-dihydrodiol (DMBA-t-3,4-diol) in our coculture system (Figure 4C-4E). Switching to DMBA was necessary because the active MCA-metabolite is not available. Following incubations of DMBA with wt PMs elicited a DNA damage response in fibroblasts, similarly to MCA. The γH2AX signal was largely attenuated when using macrophages from myeloid *Hif-1α^LysM−/−^* mice (Figure 4C, 4D, Supplementary Figure 6B). However, the metabolite DMBA-t-3,4-diol was equally potent in causing a DNA damage response when incubated with either wt or *Hif-1α^LysM−/−^* PMs (Figure 4C, 4E, Supplementary Figure 6C). This underscores the role of macrophage PAH-metabolism, dependent on HIF-1, for a DNA damage response in fibroblasts. To verify the relevance of CYP1A1 and CYP1B1 in this set up, we employed the CYP inhibitor α-naphtoflavone (α-NF). Co-stimulation of PMs with MCA and α-NF decreased H2AX phosphorylation compared to MCA-stimulation alone (Figure 4F, 4G, Supplementary Figure 6D). α-NF reduced H2AX phosphorylation to a level comparable to that seen in cocultures of NIH3T3 cells with PMs lacking *Hif-1α*. To summarize, formation of a DNA damaging PAH-metabolite demands HIF-1α in macrophages and is correlated to diminished CYP1A1 and CYP1B1 expression as well as reduced DNA damage.

### The loss of HIF-1α decreases ARNT levels in macrophages

**Figure 5:**
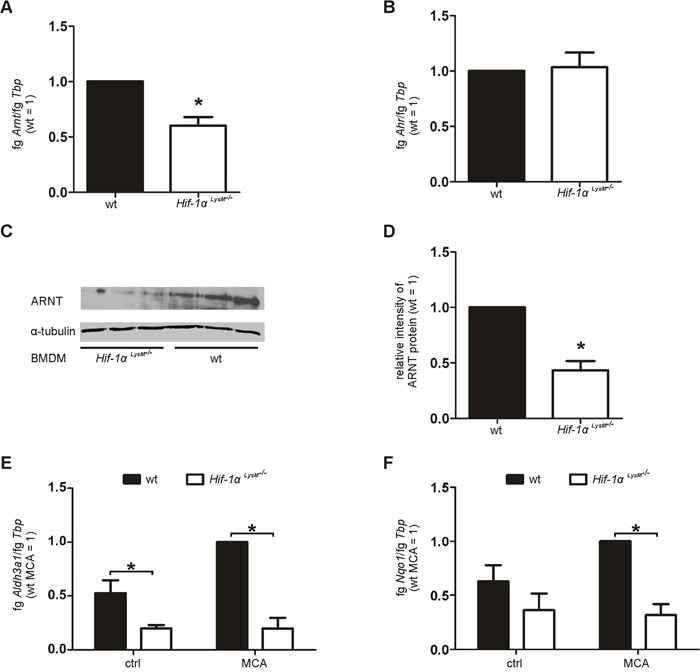
Reduced *Arnt* expression in *Hif-1α^LysM−/−^* macrophages **A.** *Arnt* and **B.** *Ahr* mRNA expression in PMs isolated from wt and *Hif-1α^LysM−/−^* mice assessed by qPCR. mRNA levels were normalized to *Tbp*. The ratio of *Arnt* or *Ahr* and *Tbp* in wt mice was set to 1. Values are the mean ± SEM of n = 4 (each n contains 3 mice/genotype). * P < 0.05 compared to wt. **C.** Representative Western analysis of ARNT protein in BMDMs isolated from wt and *Hif-1α^LysM−/−^* mice. Tubulin served as a loading control. **D.** Quantification of ARNT protein expression. To quantitate the ARNT protein, band intensity of each experiment (3 mice per genotype) was measured. The medium intensities of wt BMDMs were set to 1 and compared to the medium intensities of *Hif-1α^LysM−/−^* BMDMs. Experiment including quantification was performed three times. **E.** *Aldh3a1* and **F.** *Nqo1* mRNA expression in PMs isolated from wt and *Hif-1α^LysM−/−^* mice, stimulated with MCA or DMSO (ctrl) for 8 h assessed by qPCR. mRNA levels were normalized to *Tbp*. The ratio of *Aldh3a1* and *Tbp* in wt mice after stimulation with MCA was set to 1. Values are the mean ± SEM of n = 4 (each n contains 3 mice/genotype), * P < 0.05.

Next, we questioned whether HIF-1 directly regulates CYP1A1/CYP1B1. Therefore, *Cyp1a1* and *Cyp1b1* expression was analyzed in isolated PMs under hypoxic conditions or following their exposure to the HIF-stabilizer dimethyloxaloylglycine (DMOG). Macrophages failed to enhance *Cyp1a1* or *Cyp1b1* expression after 16 hours hypoxia (1% O_2_) or DMOG-stimulation (Supplementary Figure 7A, 7B). Regulation of CYP1A1 also remained unaffected by the loss of myeloid Hif-2α (Supplementary Figure 7A). Furthermore, treatment of cocultures with the specific PHD inhibitor IOX2 did not alter DNA damage responses in fibroblasts (Supplementary Figure 5D, 5E). As these data exclude a direct regulation of CYPs by HIF-1, we assumed that HIF-1α indirectly regulates CYP enzymes by affecting the AhR/ARNT pathway. Indeed, expression of *Arnt* mRNA was significantly reduced in macrophages of *Hif-1α^LysM−/−^* mice, whereas *Ahr* mRNA levels remained unchanged (Figure 5A, 5B). A decrease in *Arnt* mRNA expression lowered the amount of the ARNT protein in macrophages with a knockout of *Hif-1α* (Figure 5C, 5D, Supplementary Figure 7C, 7D). Aldolase 3A1 (*Aldh3a1*) and NADPH:quinone oxidoreductase 1 (*Nqo1*) mRNA, known AhR/ARNT target genes, were also significantly downregulated in *Hif-1α^LysM−/−^* PMs (Figure 5E, 5F). Similar to the behavior of CYPs, *Aldh3a1* expression was not only impaired after MCA-stimulation but also at basal level.

To test a direct regulation of ARNT by HIF-1, we analyzed the expression of *Arnt* following HIF-activation with hypoxia and DMOG. *Arnt* levels were not significantly affected by HIF activation (Supplementary Figure 7E) thus, excluding a direct regulation of ARNT by HIF-1. Further, we tested if the knockout of *Hif-1α* alters *Arnt* mRNA stability. Therefore, primary macrophages were time-dependently treated with actinomycin D (actD). A loss of *Hif-1α* did not alter *Arnt* mRNA stability (Supplementary Figure 7F). These findings suggest that metabolic activation of MCA is attenuated in *Hif-1α^LysM−/−^* macrophages, likely as a consequence of reduced ARNT transcription.

### Metabolic MCA-activation is attenuated by a knockdown of ARNT

**Figure 6:**
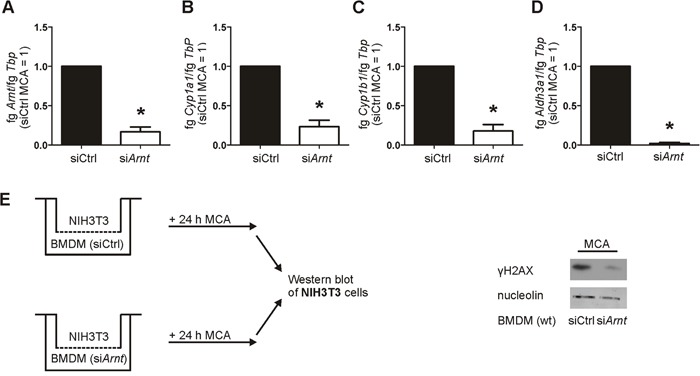
Reduced *Arnt* expression impairs AhR-mediated gene expression and DNA damage **A.** *Arnt*, **B.** *Cyp1a1*, **C.** *Cyp1b1*, and **D.** *Aldh3a1* mRNA expression in bone marrow-derived macrophages (BMDMs) transiently transfected with siRNA against *Arnt* (si*Arnt*) or control siRNA (siCtrl), followed by stimulation with MCA for 8 h, assessed by qPCR. mRNA levels were normalized to *Tbp*. The ratio of *Arnt, Cyp1a1*, *Cyp1b1*, or *Aldh3a1* and *Tbp* in siCtrl BMDMs after MCA-stimulation was set to 1. Values are the mean ± SEM of n = 4 (each n containing 3 mice/genotype), * P < 0.05 compared to siCtrl MCA. **E.** Illustration of the coculture of NIH3T3 cells with wt BMDMs that were transfected with siRNA against *Arnt* (si*Arnt*) and control siRNA (siCtrl) and incubated with MCA for 24 h. Representative Western analysis of phosphorylated γH2AX in NIH3T3 cells from 4 experiments. Nucleolin served as a loading control.

To verify the crucial role of ARNT in MCA-metabolism, we used an siRNA approach to knock down *Arnt*. Murine macrophages were isolated from wt mice and transiently transfected with siRNA targeting *Arnt* (si*Arnt*) compared to a non-targeting siRNA control (siCtrl). As it was not possible to transfect PMs, we used bone marrow-derived macrophages (BMDMs) for the transient knockdown of *Arnt*. Silencing of *Arnt* was confirmed at the mRNA level, showing a reduction of 83% compared to siCtrl BMDMs (Figure 6A). Following treatments with MCA, cells harboring a knockdown of *Arnt* showed a reduced expression of the metabolizing enzymes *Cyp1a1*, *Cyp1b1*, and *Aldh3a1* (Figure 6B, 6C, 6D) compared to siCtrl-treated BMDMs. The impact of the *Arnt* knockdown on DNA damage was analyzed with our coculture assay. Similar to coculture experiments with PMs of *Hif-1α^LysM−/−^* mice, phosphorylation of γH2AX was reduced in NIH3T3 cells following their coculture with si*Arnt* BMDMs in the presence of MCA (Figure 6E). These data link the loss of *Hif-1α*, decreased ARNT expression and attenuated CYP1A1 and CYP1B1 induction. Reduced biotransformation of MCA and tumor development in myeloid *Hif-1α^LysM−/−^* mice is thus explained by diminished ARNT expression and the subsequently reduced metabolic activation of PAHs by CYP1A1 and CYP1B1.

## DISCUSSION

Although the role of myeloid HIF-1α and HIF-2α during inflammation is well described their impact on carcinogenesis is less well understood [30, 34]. In the oncogene-driven PyMT model, tumor latency was unaffected, while tumor burden was non-significantly reduced in both knockout strains. This corroborates earlier data in PyMT mice with a myeloid-specific *Hif-1α^LysM−/−^* knockout, demonstrating no influence on tumor initiation but a reduced tumor mass [29]. Mechanistically, a loss of *Hif-1α* in macrophages attenuated their ability to suppress T-cell proliferation and activation *in vitro* thus, favoring cell death at early stages of PyMT tumor progression, assumingly lowering tumor mass *in vivo*. In this model the role of myeloid HIF-2α is unexplored. We noticed a slightly decreased tumor burden at the end of the experiment (week 20), pointing to a tumor-promoting ability of HIF-2α in the PyMT tumor model. In a murine melanoma model, activation of macrophage HIF-2α potentiates tumor-suppressive effects of GM-CSF and enhances survival of mice, while effects of HIF-activation in the absence of GM-CSF during tumor development remain unclear [35]. In a xenograft model of Lewis Lung carcinoma (LLC) and B16BL6 melanoma cells activation of both HIF-isoforms, by ablating PHD2 in macrophages and T-cells, attenuated tumor development due to altered cell death and proliferation of tumor cells [36]. These findings suggest an organ- and context-specific effect of HIF on spontaneous tumor development.

In contrast to minor effects in the oncogene-driven cancer model the loss of *Hif-1α* in myeloid cells almost completely attenuated tumor development in the chemical MCA-carcinogen driven model. The model is characterized for its inflammatory component during early tumor formation and thus, interfering with inflammation lowers the tumor incidence [31, 37]. Consistent with these data, we noticed the accumulation of neutrophils and macrophages in close proximity to lipid droplets, formed at the injection sites, but no dependency on HIF-1 towards these signs of inflammation. These observations are supported by the notion that inflammatory cytokines are equally produced in wt and *Hif-1α^LysM−/−^* mice, suggesting that early inflammatory markers do not account for differences in tumor progression.

PAHs are prevalent carcinogens that require biotransformation and thus, metabolic activation by CYP1A1 and CYP1B1 [6, 38-40]. These CYPs are not only abundant in the liver but are also expressed in extra hepatic tissue and are crucial for the DNA damage response of PAHs in these tissues [39]. Our data suggest that myeloid cells in the skin contribute to metabolic PAH activation and subsequent DNA damage in fibroblasts, thereby initiating tumorigenesis. Consistent, targeting or depleting myeloid cells *in vivo* revealed the importance of these cells for tumor induction in different cancer types before [41, 42]. Our data confirm and extend a recent publication identifying Langerhans cells as an essential component for CYP-mediated PAH-metabolism in the 7,12-dimethylbenz[a]anthracene (DMBA) two-stage cutaneous chemical carcinogenesis model [9]. These observations and our data link biotransformation and activation of PAH carcinogens in the skin to cells of the myeloid compartment. Depleting myeloid cells via clodronate to prove this assumption *in vivo* is technically not possible in long-term experiments such as the MCA-model. As Langerhans cells also express Lysozyme M (LysM) [43] and thus, are depleted of HIF-1 in our conditional knockout mouse as well, it can be speculated that these tissue-associated cells also relay on HIF-1 to enhance chemical carcinogenesis via PAH-metabolism. Besides pointing to myeloid cells in the skin as a necessary component for PAH-metabolisms, our data provide evidence for a role of HIF-1 but not HIF-2 in this process and furthermore emphasize that downregulation of CYPs in myeloid cells is sufficient to attenuate tumor outgrowth in the skin. Replacing the PAH DMBA by its DNA-damaging metabolite DMBA-trans-3,4-dihydrodiol (DMBA-t-3,4-diol), we could restore reduced DNA damage in NIH3T3 cells cocultured with *HIF-1α^LysM−/−^* PMs, thereby confirming limited metabolic activation as the crucial parameter for attenuated DNA damage. Upregulation of CYP1A1 and CYP1B1 by PAH requires dimerization of AhR with ARNT [3]. We noticed reduced *Arnt* expression in *Hif-1α* knockout macrophages as a likely explanation for the attenuated CYP induction. Previous studies already suggested interactions between the AhR/ARNT and HIF-α/ARNT pathways. Studying the competition between HIF-1α and AhR for ARNT revealed attenuated AhR/ARNT target gene expression under hypoxic conditions [5, 22, 44]. This goes along with studies confirming a higher affinity of HIF-1α compared to AhR for the constitutively expressed ARNT [21]. Moreover, upregulation of ARNT by hypoxia or HIF-stabilizers has been described, although molecular mechanisms remain obscure [45-50]. Interestingly, reduced *Arnt* mRNA levels under normoxic conditions *in vitro* in Min6 cells after a knockdown of HIF-1α as well as *ex vivo* in islets from *Hif-1α* null-mice were shown, without characterizing potential consequences [51]. To date, the complex interaction of HIF-α isoforms, ARNT, and AhR is not fully understood. So far we excluded an altered mRNA stability and direct regulation of ARNT by HIF-1, which may point to transcriptional regulation of ARNT in *Hif-1α^LysM−/−^* mice. To mechanistically explain how a loss of *Hif-1*α in macrophages downregulates ARNT future investigations are required.

The loss of *Hif-2α* left tumor initiation unaffected, but accelerated tumor development and enhanced tumor burden in the MCA model. This may point to a tumor-repressing ability of HIF-2α. The impact of HIF-2α on tumorigenesis was already analyzed in a model of murine chemically-induced colitis-associated cancer (CAC) and hepatocellular carcinoma (HCC). Impaired tumor cell proliferation and progression in the CAC model after the myeloid loss of *Hif-2α* was attributed to reduced TAM infiltration with no effect on HCC progression [30]. We neither observed reduced immune cell infiltration with a knockout of *Hif-2α*, nor differences in vascularization, which implied tumor- and/or organ-specific effects of myeloid HIF-2α.

Collectively, the loss of *Hif-1α* in myeloid cells attenuated CYP-mediated metabolic activation of PAHs, subsequent *in vivo* and *ex vivo* DNA damage, and reduced tumor development. PAHs are part of tobacco smoke and air pollutants. [52-55]. An increasing potential of carcinogens as part of air pollution causes most cancer deaths, besides triggering cardiovascular diseases [1]. Others have already revealed the need for nonepithelial stromal cells to activate PAH mutagens [9, 56]. The marked resistance of myeloid cells with a knockout of *Hif-1α* to chemical carcinogenesis establishes the capacity of HIF-1 to substantially enhance toxicity of environmental agents. Our data are consistent with a cooperative carcinogenicity scenario, where CYP1A1/CYP1B1 metabolize MCA to a DNA damaging agent, which is delivered to adjacent fibroblasts to initiate DNA damage and start tumorigenesis. These studies open the possibility that HIF-1 in resident macrophages is a prerequisite for PAH-induced mutations and tumor development within other epithelial tissues, contributing to the risk of colon, lung, or genitourinary carcinomas. Further studies need to prove whether HIF-1 specific inhibitors can be used to prevent chemically-induced skin or lung carcinoma.

## MATERIALS AND METHODS

### Myeloid-specific knockout of HIF-1α and HIF-2α

*Hif-1α*^flox/flox^/lysozyme M Cre (LysMCre) mice (Hif-1α^*LysM-/−*^) were generated and kindly provided by Prof. R.S. Johnson. *Hif-2α*^flox/flox^ mice were obtained from Prof. M.C. Simon and crossed to LysMCre mice to obtain myeloid-specific *Hif-2α* knockout mice (Hif-2α^*LysM-/−*^ ). Mice were established as 129 Sv x C57BL/6 mice containing floxed sites flanking exon 2 of the *Hif-1α* or *Hif-2α* gene and were backcrossed into the C57BL/6 background. *Hif-1α/2α*^flox/flox^/LysMCre (Hif-1α/2α^*LysM-/−*^ ) mice were bred by crossing *Hif-1α*^flox/flox^/LysMCre mice containing loxP sites around exon 2 of *Hif-2α*. Mice having flanked loxP sites but were negative for the Cre recombinase served as controls (wt mice).

### PyMT breast cancer model

For details see Supplementary Methods.

### MCA induced fibrosarcomas

Male mice were subcutaneously (s.c.) injected in the right thigh with 100 μg MCA dissolved in 100 μl corn oil (Sigma-Aldrich, Steinheim, Germany). Tumor progression in mice as well as mice weight was evaluated twice weekly by palpation [29, 31]. Tumors developed within week 8-20 and mice were sacrificed after 21 weeks. Tumor tissue was extracted and weighed. Part of the tumor tissue was fixed in 4% paraformaldehyde (PFA) or zinc fixation buffer for immunohistochemistry (IHC), while remaining tissue was prepared for flow cytometry analysis. For studies at early time points, mice were injected with the indicated MCA concentration in one thigh and with 100 μl corn oil as a control in the other thigh. Skin tissue around the injection site with a diameter of approximately 1.5 cm was isolated 5 days after injection and either fixed in 4% PFA or frozen in liquid nitrogen for RNA isolation.

### Flow cytometry

For details see Supplementary Methods.

### Isolation of primary mouse macrophages

Peritoneal macrophages (PMs) and bone marrow-derived macrophages (BMDMs) were isolated as described previously [57].

### Cell culture and incubation procedures

NIH3T3, bone-marrow-derived macrophages (BMDMs), and peritoneal macrophages (PMs) were cultured in Dulbecco's Modified Eagle's Medium DMEM (high glucose) supplemented with 10% heat-inactivated fetal calf serum (FCS), 100 U/ml penicillin, and 100 μg/ml streptomycin (all from PAA). Cells were kept at 37°C in a humidified atmosphere with 5% CO_2_. BMDMs were differentiated for 7 days using 20 ng/ml macrophage colony-stimulating factor (M-CSF, Peprotech, Hamburg, Germany). PMs were cultured for 2 days before treatment. For hypoxic exposure cells were incubated either with 1% O_2_ in a hypoxia workstation InVivo400 (Ruskinn Technology, Leeds, UK) or 1 mM dimethyloxalylglycine (DMOG; Alexis Biochemicals, Lörrach, Germany) for 16 h. For RNA analysis, primary macrophages were incubated with MCA (5 μg/ml) or dimethylsulfoxide (DMSO) as a control for 8 h. To analyze mRNA stability, differentiated BMDMs were treated with 2.5 μg/ml actinomycin D (Sigma-Aldrich) for times indicated.

### siRNA transfection

For transfection, isolated BMDMs were differentiated with 20 ng/ml M-CSF for 1 week and transfected afterwards with 150 nM SMARTpool ON-TARGET plus si*Arnt* or ON-TARGET plus Non-targeting pool siCtrl (both Thermo Scientific, Karlsruhe, Germany) using Hiperfect transfection reagent (Quiagen, Hilden, Germany). Therefore, BMDMs were incubated in medium without FCS containing siRNA and Hiperfect. After 6 h, medium containing M-CSF (final concentration of 20 ng/ml) was added and after 24 h cells were stimulated with MCA (5 μg/ml) or dimethylsulfoxide (DMSO) as a control for 8 h.

### Fibroblast-macrophage cocultures

PMs or BMDMs were isolated as described and plated into the lower compartment of transwell inserts (6-well plates), while NIH3T3 fibroblasts were seeded in the upper well of the inserts (0.1 μm pore size). The following day, cells were stimulated with MCA (5 μg/ml), 7,12-dimethylbenz[a]anthracene (DMBA: 2 μg/ml), DMBA-trans-3,4-dihydrodiol (DMBA-t-3,4-diol: 2 μg/ml) or DMSO (0.1%) for 24 h. Afterwards, NIH3T3 cells were harvested for Western analysis. Alternatively, cells were prestimulated for 1 h with 10 nM α-naphtoflavone (α-NF) (Sigma-Aldrich), followed by the addition of MCA for 24 h.

### RNA isolation

For RNA preparation from skin, tissue was obtained as described above, frozen and dissected by an IKA^®^T25 digital UltraTurrax^®^ (IKA^®^-Werke GmbH & Co. KG, Staufen, Germany) in peqGold (Peqlab, Erlangen, Germany). RNA from skin and cells was isolated and qPCR was performed as described before [58].

### Quantitative real time PCR (qPCR)

For details see Supplementary Methods.

### Western analysis

As PMs yield not enough protein for Western analysis, the protein level of ARNT was examined in BMDMs instead of PMs. Cells were washed in PBS, lysed in cell lysis buffer (6,65 M urea, 10% glycerol, 1% SDS, 10 mM Tris/HCl, pH 6.8; pH adjusted to 7.4) and sonicated following centrifugation (16000 g, 10 min). After quantification of the protein amount using a protein assay kit (Bio-Rad) 60 μg protein was loaded on 15% SDS-polyacrylamide gels and blotted onto a Immobilon-FL polyvinylidene difluoride (PVDF)-membrane (Millipore). Western analysis of ARNT was performed with 100 μg protein on a 10% SDS-polyacrylamide gel. Membranes were incubated using antibodies against phospho-Histone H2AX (γH2AX: Millipore), ARNT (Abcam), α-tubulin (Sigma-Aldrich), actin (Sigma-Aldrich), and nucleolin (SantaCruz, Heidelberg, Germany) overnight at 4°C. Blots were incubated with the appropriate secondary antibodies and detected using either Enhanced Chemiluminescence (ECL) or the Odyssey infrared imaging system (Li-COR Biosciences, Bad Homburg, Germany). ARNT blots were quantified by ImageJ 1.3 (NIH).

### Immunohistochemistry

Tissue samples were dehydrated in alcohol (Sigma-Aldrich) and embedded in paraffin. Sections of 4 μm were deparaffinized in xylene (Sigma-Aldrich) and rehydrated in alcohol. For antigen retrieval, sections were boiled for 12 min in TRS buffer (DAKO, Hamburg, Germany). Endogenous Avidin and Biotin were blocked by using a biotin-blocking system (DAKO). For staining, a Catalyzed Signal Amplification (CSA) kit (DAKO) was used. Primary antibodies were incubated at 4°C overnight. Primary antibodies used: anti-mouse F4/80 (eBioscience, Frankfurt, Germany), anti-CD31 (Chemicon International, Temecula, CA, USA), anti-mouse Ym1 rabbit polyclonal antibody (StemCell Technologies, Grenoble, France), anti-phospho-Histone H2AX (Millipore, Darmstadt, Germany; Abcam, Cambridge, UK), CYP1A1 (Abcam, Cambridge, UK), and Ly6B.2 allogenic antibody (AbD Serotec, Puchheim, Germany). Afterwards, corresponding biotinylated secondary antibodies (biotinylated anti-rat from DAKO and biotinylated anti-rabbit from Axxora, Loerrach, Germany) were applied. For color development Diaminobenzidin (DAB) (contained in the CSA kit) was added as a substrate to the peroxidase-coupled secondary antibody. Sections were analyzed using the microscope Axioskop 40 (Carl Zeiss AG, Göttingen, Germany). CD31 positive signals were quantified using HistoQuest 2.X (Tissue Gnostics, Wien, Austria).

### Liquid chromatography coupled to tandem mass spectrometry (LC-MS/MS)

For LC-MS/MS detection, PMs were incubated with MCA or DMSO for 8 h, followed by collection of cell supernatants. To determine analyte retention times and mass fragmentation, the standard 3-methylcholanthrene-cis-11,12-dihydrodiol (11,12-diOH-3-MCA) was obtained from MRIGlobal, Kansas City, MO, USA. For sample extraction, 600 μl of each culture media was mixed with 50 μl standard/methanol and 20 μl internal standard solution (10 μg/ml of 1-pyrenol in methanol (Sigma-Aldrich)) and extracted twice with ethyl acetate (1 ml and 600 μl). The organic supernatant was evaporated under a gentle stream of nitrogen at 45°C following reconstitution in 50 μl methanol. Samples were injected into the liquid chromatography-electrospray ionization-tandem mass spectrometry (LC-ESI-MS/MS). The system consisted of a hybrid triple quadrupole-ion trap mass spectrometer QTrap 4000 (AB Sciex, Darmstadt, Germany) equipped with a Turbo-V-source, an Agilent 1100 chromatographic system (Agilent, Waldbronn, Germany), and an HTC Pal autosampler (CTC Pal, Zwingen, Switzerland). The system was operated in positive ionization mode at 5500 V and 400°C in MS^3^ scan modus.

For chromatographic separation a Max-RP column (150 × 2 mm, Phenomenex, Aschaffenburg, Germany) was used with a linear gradient at a flow rate of 300 μl/min. Mobile phase A was water/ammonia (100:0.01, v/v) and mobile phase B was methanol/ammonia (100:0.01, v/v). Total run times were 40 min and sample volumes for injection were 20 μl. Calibration curves ranged from 0.1 to 10 ng/mL for 11,12-diOH-3-MCA. Samples were quantified using the internal standard method and the software Analyst 1.4 (AB Sciex, Darmstadt, Germany).

### Statistical analysis

All experiments were performed at least 3 times and results are expressed as means ± SEM. Graphs showing tumor development were generated with Kaplan-Meier analysis and compared using the log-rank test. Student's t-test was performed to evaluate differences between wt and *Hif-1α^LysM−/−^* samples and multiple-group comparisons were calculated after analysis of variance (ANOVA) and Bonferroni's test. Differences at p < 0.05 were considered statistically significant.

### Study approval

Animal experiments were approved by local authorities (approval number F144/05) and performed following the guidelines of Hessian animal care and use committee.

## SUPPLEMENTARY MATERIALS FIGURES AND TABLES


