# Supplementary material for: Loss of HIF-1β in macrophages attenuates AhR/ARNT-mediated tumorigenesis in a PAH-driven tumor model
Source: Oncotarget. 2016 Mar 23;7(18):25915–29. doi: 10.18632/oncotarget.8297 (PMC5041954; doi:10.18632/oncotarget.8297)
Supplement: Supplementary file 1 [file oncotarget-07-25915-s001.pdf]

SUPPLEMENTARY DATA

SUPPLEMENTARY MATERIALS

PyMT breast cancer model

Mice with the myeloid-specific knockout of *Hif-1α*, *Hif-2α*, and *Hif-1α/2α* as well as wt mice were crossed with mice expressing the PyMT oncogene under the Mouse Mammary Tumor Virus promoter (MMTV)-PyMT mice. All PyMT mouse strains were backcrossed for at least six generations. Tumor development in female mice was assessed twice weekly. Mice were sacrificed after 20 weeks and tumor tissue was analyzed.

Flow cytometry

Tumor cells were minced into small pieces with a scalpel and digested either with accutase and 1 U/ml DNAse I (Promega, Mannheim, Germany) in DMEM (1:1) (PAA, Cölbe, Germany) for 30 minutes to stain the leucocyte panel or 20 minutes with 3 mg/ml Collagenase IA (Sigma-Aldrich, Steinheim, Germany) and 1 U/ml DNAse I (Promega) in DMEM for the myeloid panel at 37°C. Digested cells were homogenized with a MediMachine (BD Biosciences, Heidelberg, Germany). Red blood cells were lysed (red blood cells lysis buffer) and filtered through a 70 µm nylon mesh (BD Biosciences, Heidelberg, Germany). Cells were blocked in FACS tubes with BD Fc Block (BD Biosciences) for 20 minutes followed by staining with fluorochrom-conjugated antibodies for 15 minutes and measurement on a LSRII/Fortessa flow cytometer (BD Biosciences). Samples were quantified with FACSDiva Software (BD Biosciences).

For FACS analysis of leucocytes subsets in the tumor we used the following antibodies: anti-CD45-Vioblue (Miltenyi Biotech, Bergisch Gladbach, Germany), anti-CD3-APC (BD Biosciences), anti-CD4-V500 (BD Biosciences), anti-CD8a-AlexaFluor 700 (BD Biosciences), anti-CD11b-eFluor 605 (eBioscience, San Diego, CA), anti-CD19-APC-H7 (BD Biosciences), anti-CD25-Pe-Cy7 (BD Biosciences), anti-CD49b-PE (Miltenyi Biotech) and anti-GITR-FITC (BD Biosciences).

For FACS analysis of myeloid cells in the tumor the following antibodies were used: anti-CD45-Vioblue (Miltenyi Biotech), anti-CD11b-eFluor605 (eBioscience), anti-CD11c-AlexaFluor700 (BD Biosciences), CD49b-PE (Miltenyi Biotech), anti-F4/80-PeCy7 (eBioscience), anti-mPDCA-1-FITC (Miltenyi Biotech), anti-MHCII-APC (Miltenyi Biotech), anti-Ly6C-PerCP-Cy5.5 (BD Biosciences) and anti-Ly6G-APC-Cy7 (BD Biosciences).

For cell cycle analysis, NIH3T3 cells were cocultured with/without wt or *Hif-1α* $^{LysM-/-}$ peritoneal macrophages and stimulated with MCA for 24 h as described in the Method section. Cells were harvested in PBS, lysed in PBS containing 1.1 g/l glucose and 0.5 mM EDTA (PBS^+) and centrifuged again. Cell pellets were incubated in PBS^+ supplemented with 50 µg/ml RNase for 15 minutes followed by staining with 10% NP-40 and 7-AAD (BD Biosciences) for 15 minutes. Stained cells were measured by LSRII/Fortessa flow cytometer (BD Biosciences). Samples were quantified with FACSDiva Software (BD Biosciences).

To follow apoptosis, cells were detached with accutase, stained with Annexin V (ImmunoTools, Friesoythe, Germany) and 7-AAD (BD Biosciences) for 20 minutes and measured by LSRII/Fortessa flow cytometer. Samples were quantified with FACSDiva Software. Apoptotic cells were FITC–positive, while necrotic cells were FITC-positive and PE-Cy5-positive.

Quantitative real time PCR (qPCR)

Expression of the genes was measured with SYBRgreen fluorescein mix (Thermo Scientific) in a CFX96 Realtime PCR Detection System (Bio-Rad, Munich, Germany) according to manufacture instructions. Primers sequences are provided in Supplementary Table 1.

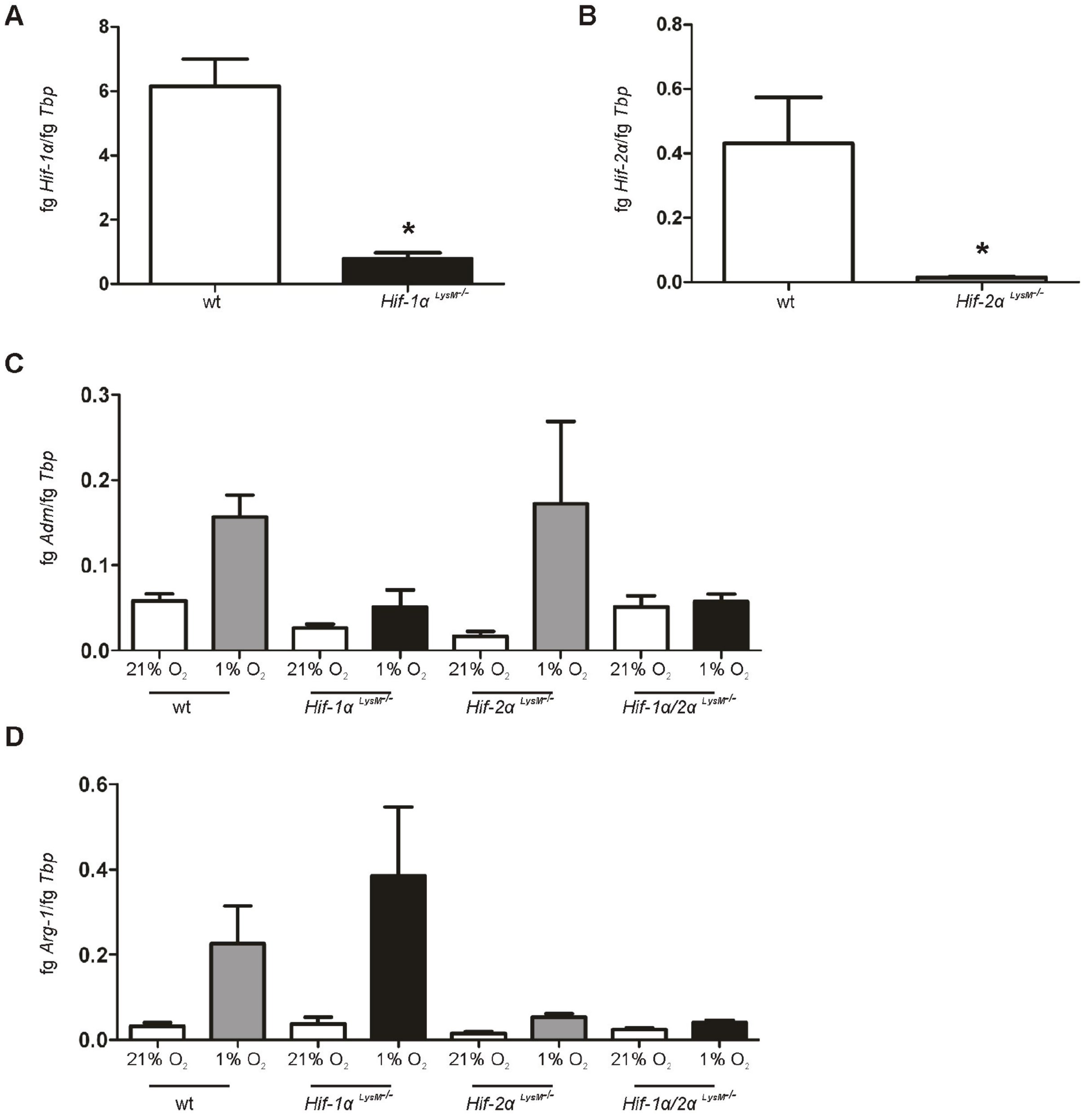

Supplementary Figure S1: Regulation of *Hif-1α*, *Hif-2α*, and its target genes after the knockdown of *Hif-1α* or *Hif-2α*. **A.** *Hif-1α* and **B.** *Hif-2α* mRNA expression in peritoneal macrophages from wt, *Hif-1α* $^{LysM-/-}$, or *Hif-2α* $^{LysM-/-}$ mice assessed by qPCR. **C.** Adrenomedullin (*Adm*) and **D.** Arginase 1 (*Arg 1)* mRNA expression in bone marrow-derived macrophages derived from wt, *Hif-1α* $^{LysM-/-}$, *Hif-2α*$^{LysM -/-}$, and *Hif-1α/2α* $^{Lys -/-}$ mice, incubated at 21% O_2 or 1% O_2 for 16 h assessed by qPCR. Values are means ± SEM of n = 4 (* $P <$ 0.05 compared to wt).

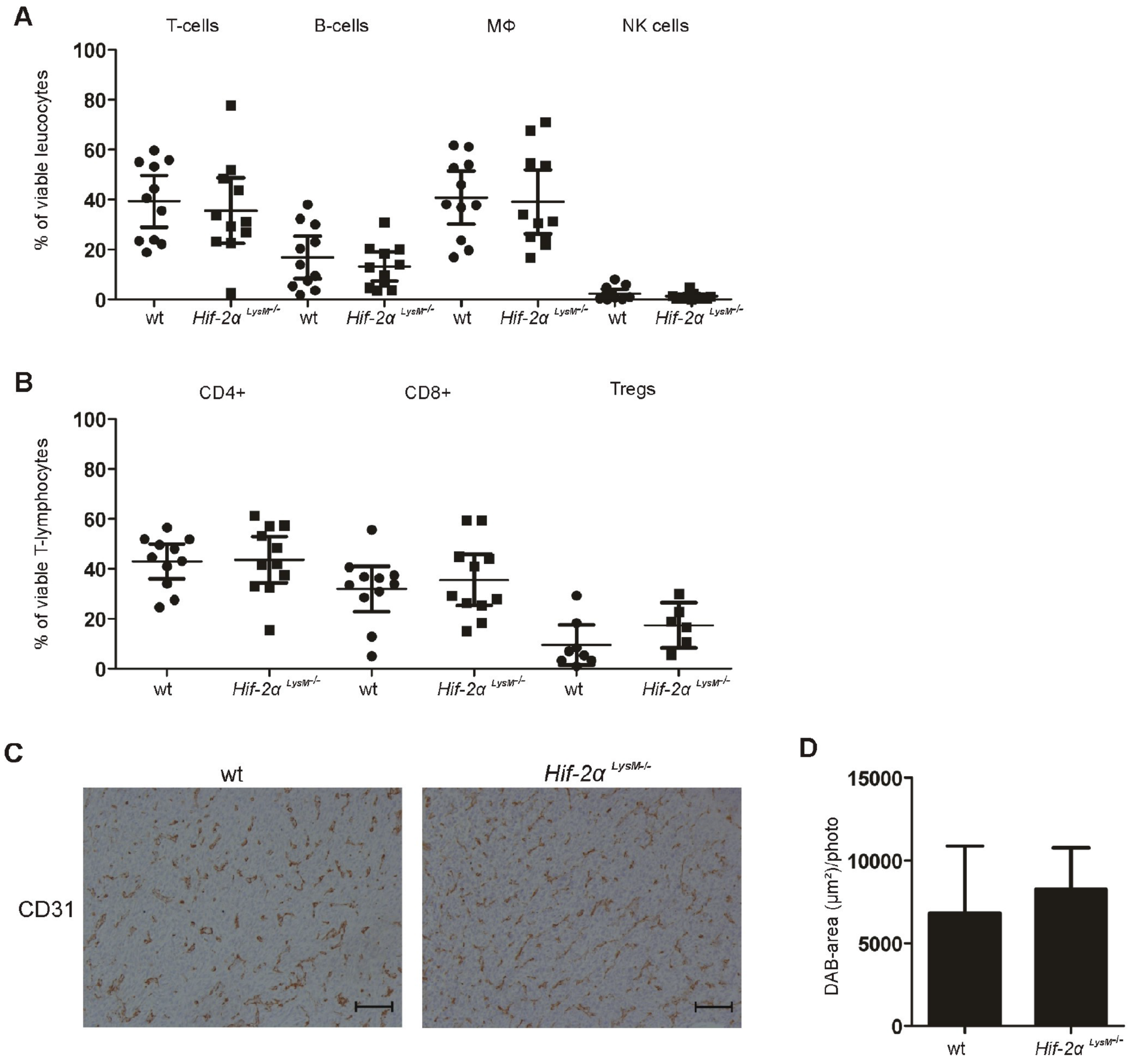

Supplementary Figure S2: Impact of macrophage HIF-2α on immune cell distribution and vascularization in MCA-induced tumors. A. Quantification of distinct cell populations related to viable leucocytes of each sample and **B.** quantification of T-cell subset populations related to the amount of T-lymphocytes of each sample in tumors of wt C57BL6/J and *Hif-2α* $^{LysM-/-}$ mice analyzed by flow cytometry. Data points represent the relative cell amount of 1 animal, with 10-11 animals per group. Each graph of the dot plots shows the median (black bars) ± SEM of n ≥ 10. **C, D.** Representative images of CD31-labeled (brown) tumors. Sections were counterstained with Mayer´s hemalaun (violet). Scale bars: 100 µm. (D) Quantification was performed by using HistoQuest. Bars are the mean ± SEM of n = 7.

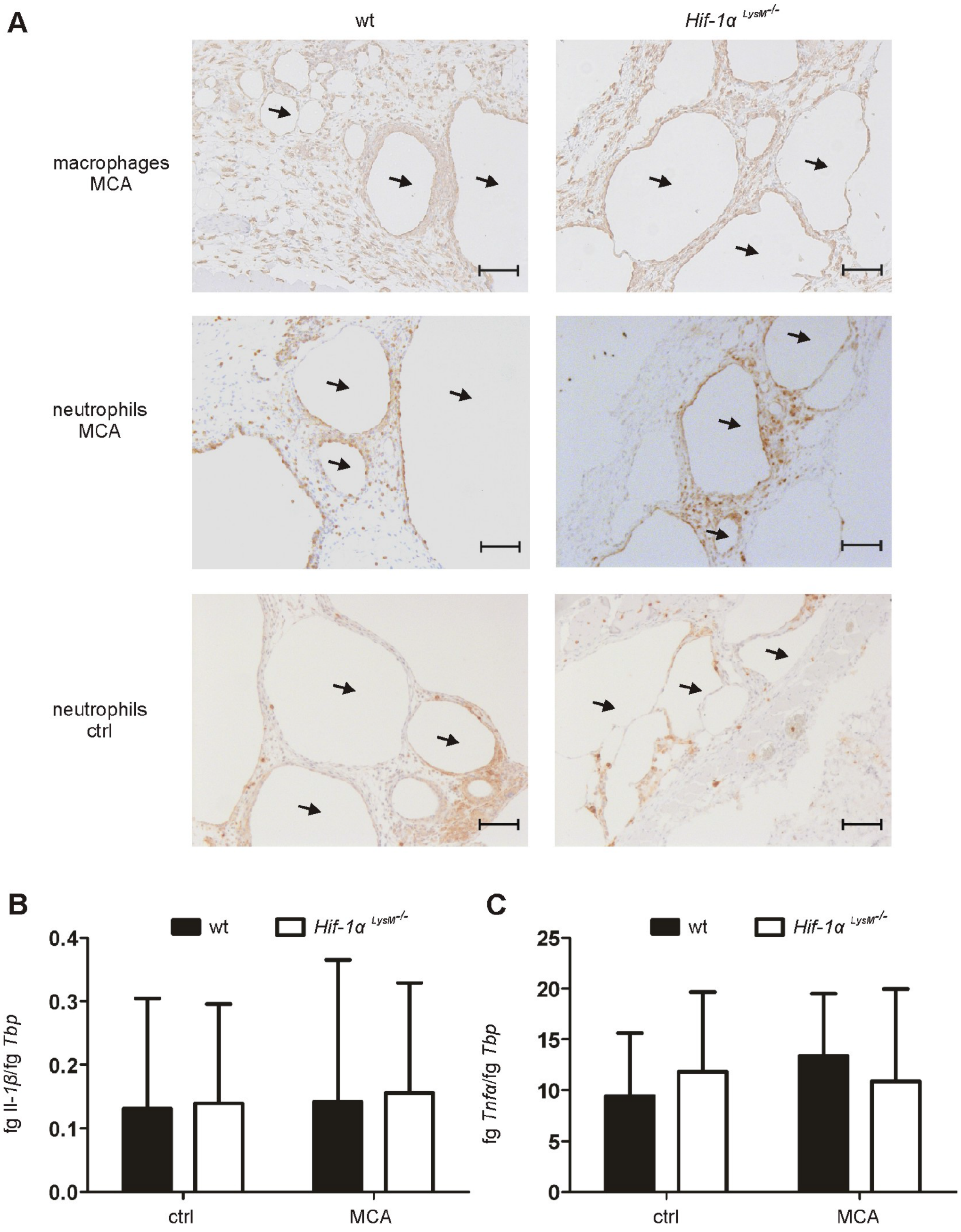

Supplementary Figure S3: Impact of macrophage HIF-1α on inflammation and DNA damage at the MCA-injection site. A. Representative immunohistochemistry of skin sections 5 days after MCA- or corn oil-injection (ctrl), stained with antibodies to detect macrophages (F4/80) and neutrophils (Ly6B.2). Sections were counterstained with Mayer´s hemalaun (violet). Arrows indicate lipid droplets. Scale bars: 100 µm. **B.** *Il-1β* and **C.** *Tnfα* mRNA expression in skin tissue in close proximity to the injection site 5 days after MCA-application. Values are means ± SEM of n = 7.

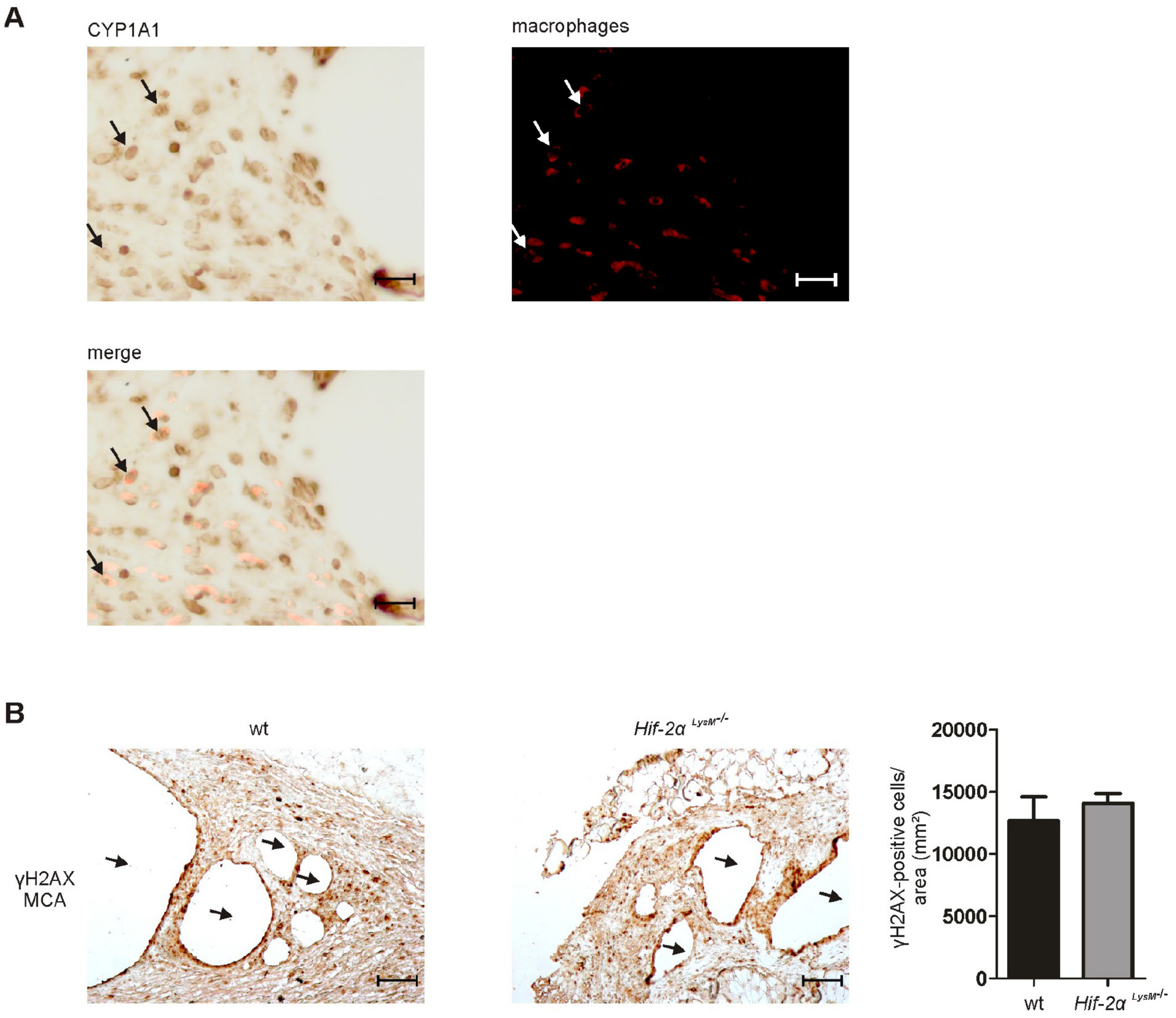

Supplementary Figure S4: CYP1A1 expression in macrophages and DNA damage in skin around the injection site. A. Representative image of a costaining (IHC) of CYP1A1 (brown) and F4/80 (red fluorescence) in skin of wt mice 5 days after MCA-injection. Arrows indicate F4/80+ cells expressing CYP1A1. Scale bars: 20 µm. **B.** Representative images and quantification of skin sections 5 days after MCA-injection, stained for γH2AX (brown) by immunohistochemistry. Arrows indicate lipid droplets. Scale bars: 100 µm. Values are the mean ± SEM of n = 3 (*Hif-2α* $^{LysM\ -/-}$)/ 4 (wt), * P < 0.05 compared to wt.

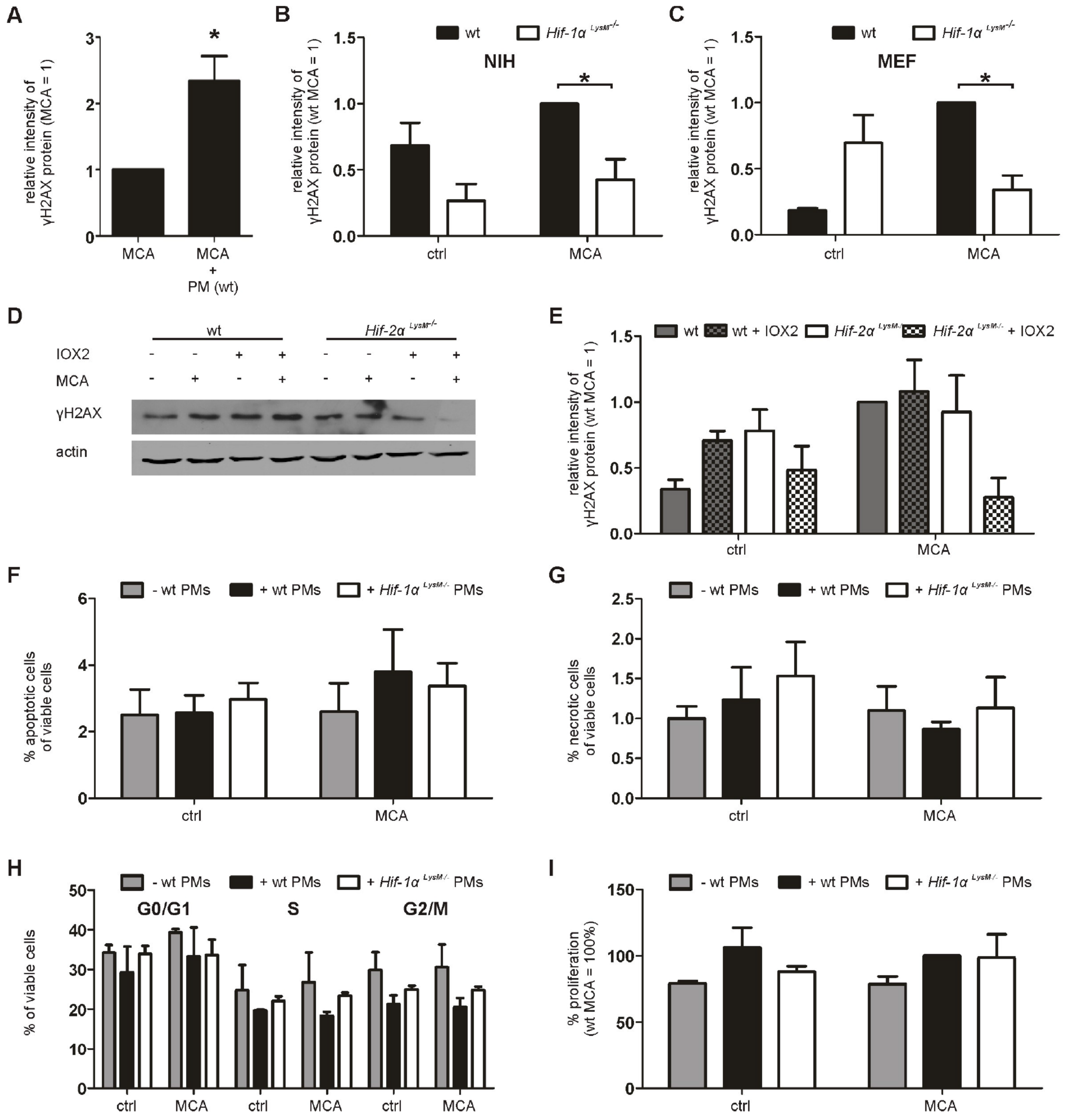

Supplementary Figure S5: Impact of macrophages and HIF on fibroblast behavior. A. Quantification of the γH2AX protein in the coculture of NIH3T3 cells and peritoneal macrophages (PMs) isolated from wt mice or in the absence of PMs stimulated with 5 µg/ml 3-methylcholanthrene (MCA) for 24 h. Values are means ± SEM of n = 4 (each n contains 3 mice/genotype). * $P < 0.05$ compared to MCA. **B.** Quantification of the γH2AX protein in NIH3T3 and in MEF **C.** cells cocultured with peritoneal macrophages (PMs) isolated from wt or *Hif-1α* $^{LysM-/-}$ mice stimulated with 5 µg/ml 3-methylcholanthrene (MCA) or DMSO (ctrl) for 24 h. Values are means ± SEM of n = 4 (each n contains 3 mice/genotype). * $P < 0.05$ compared to wt MCA. **D.** Representative Western blot and **E.** quantification of phosphorylated γH2AX in NIH3T3 cells cocultured with PMs isolated from wt or *Hif-2α* $^{LysM-/-}$ mice and stimulated with/without MCA and with or without the PHD inhibitor IOX2 (75 µM) for 24 h. Values are means ± SEM of n = 4 (each n contains 3 mice/genotype). * $P < 0.05$ compared to MCA. **F.** Apoptosis, **G.** necrosis and **H.** cell cycle distribution of NIH3T3 cells cocultured without or with PMs isolated from wt or *Hif-1α* $^{LysM-/-}$ mice stimulated with 5 µg/ml 3-methylcholanthrene (MCA) or DMSO (ctrl) for 24 h measured by flow cytometry. Values are means ± SEM of n = 3. **I.** Proliferation of cocultured or non-cocultured NIH3T3 cells as described above is measured by cell counting. Values are means ± SEM of n = 5.

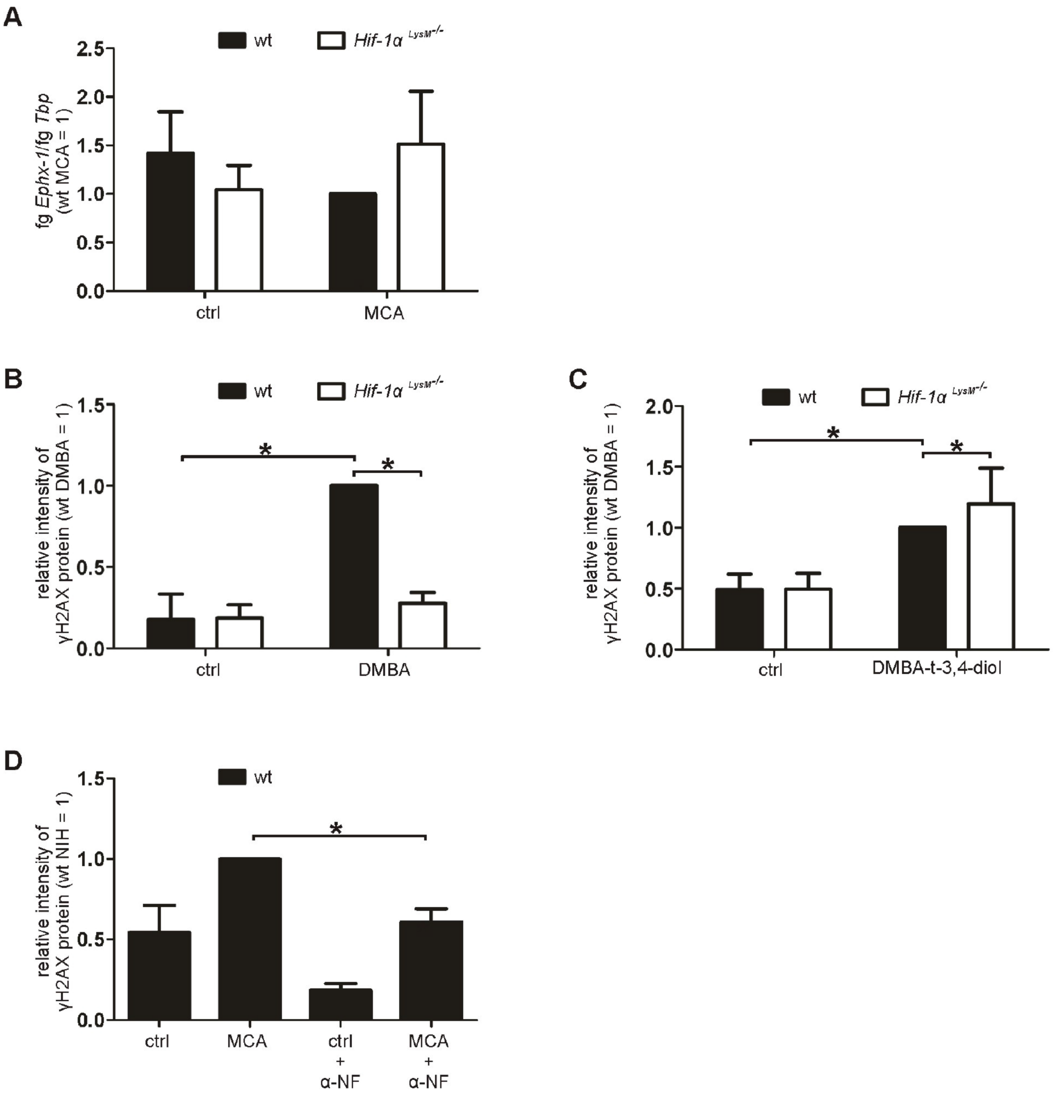

Supplementary Figure S6: Regulation of metabolizing enzymes and DNA damage after stimulation with 7,12-dimethylben[a]anthracene (DMBA) and its metabolite. A. Epoxide hydrolase-1 (*Ephx-1)* mRNA expression in peritoneal macrophages that were isolated from wt and *Hif-1α* $^{LysM-/-}$ mice, stimulated with MCA or DMSO (ctrl) for 8 h. Values are means ± SEM of n = 4 (each n contains 3 mice/genotype). Quantification of the γH2AX protein in the coculture of NIH3T3 cells with peritoneal macrophages isolated from wt or *HIF-1α* $^{LysM-/-}$ mice stimulated with **B.** 2 µg/ml 7,12-dimethylbenz[a]anthracene (DMBA), **C.** 2 µg/ml DMBA-trans-3,4-dihydrodiol (DMBA-t-3,4-diol) or DMSO for 24 h (n = 4). * P < 0.05 compared to wt DMBA. **D.** Quantification of the γH2AX signal in the coculture of NIH3T3 and wt PMs, prestimulated for 1 h with or without 10 nM α-napthoflavon (α-NF), a CYP-inhibitor, following stimulation with MCA or DMSO (ctrl) for 24 h (n = 4). * P <0.05 compared to wt MCA.

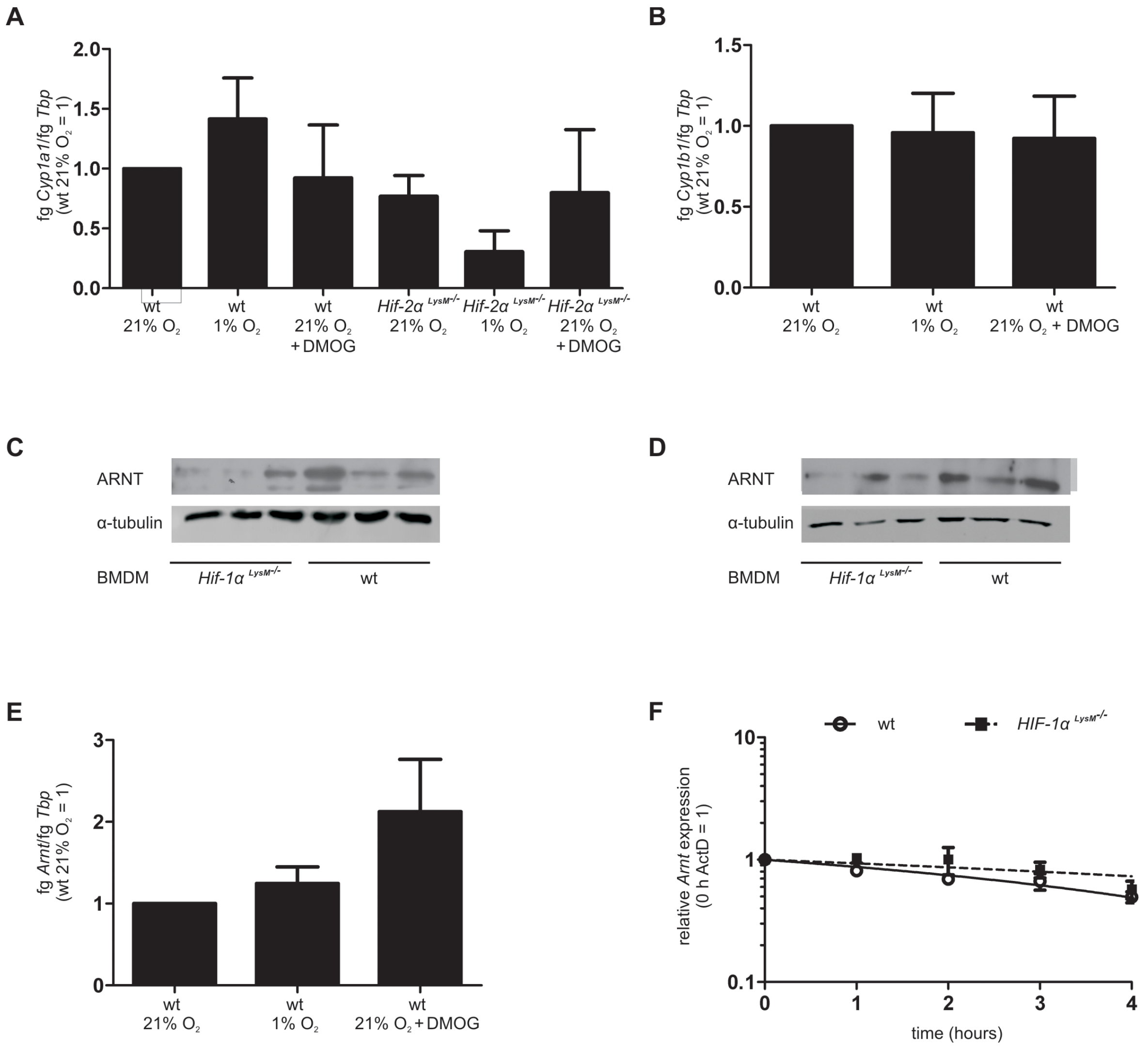

Supplementary Figure S7: ***Regulation of Cyp1a1, Cyp1b1, and Arnt.*** **A.** *Cyp1a1*, and **B.** *Cyp1b1* mRNA expression in wt and *Hif-2α* $^{LysM-/-}$ peritoneal macrophages incubated at 21% O_2, 1% O_2, or exposed to 1 mM dimethyloxalylglycine (DMOG) for 16 h assessed by qPCR. Values are the mean ± SEM of n = 4 (each n contains 3 mice/genotype). **C, D.** Representative Western analysis of ARNT protein in BMDMs isolated from wt and *Hif-1α* $^{LysM-/-}$ mice. Tubulin served as a loading control. **E.** *Arnt* mRNA expression in wt peritoneal macrophages incubated at 21% O_2, 1% O_2, or exposed to 1 mM dimethyloxalylglycine (DMOG) for 16 h assessed by qPCR. **F.** mRNA half-life and decay of *Arnt* in bone marrow-derived macrophages of wt and *Hif-1α* $^{Lys-/-}$ mice was assessed by qPCR after the treatment with 2.5 µg/ml actinomyin D (ActD) for times indicated. Each data point is the mean ± SEM of n = 6.

Supplementary Table S1: Primers used for qRT-PCR

Gene	Sequence
m*Tbp*	ctg acc act gca ccg ttg cca<br>gac tgc agc aaa tcg ctt ggg a
m*Hif-1α*	gaa atg gcc cag tga gaa aa<br>agt cta gag atg cag caa gat ctc ggc
m*Hif-2α*	cta agt ggc ctg tgg gtg at<br>gtg tct tgg aag gct tgc tc
m*Cyp1a1*	ggc cac ttt gac cct tac aa<br>cag gta acg gag gac agg aa
m*Cyp1b1*	ttc tcc agc ttt ttg cct gt<br>taa tga agc cgt cct tgt cc
m*Il-1β*	agg cca cag gta ttt tgt cg<br>gac ctt cca gga tga gga ca
m*Tnf-α*	cca ttc ctg agt tct gca aag g<br>agg tag gaa ggc ctg aga tct tat c
m*Arnt*	tgc ctc atc tgg tac tgc tg<br>gaa cat gct gct cac tgg aa
m*Ahr*	acc aga act gtg agg gtt gg<br>tct gag gtg cct gaa ctc ct
m*Aldh3a1*	ccc ctg gca ctc tat gtg tt<br>gag acc tca cca ggc aag ag
m*Arg-1*	gtg aag aac cca cgg tct gt<br>ctg gtt gtc agg gga gtg tt
m*Adm*	cgc agt tcc gaa aga agt gg<br>cca gtt gtg ttc tgc tcg tcc
m*Nqo1*	ttc tct ggc cga ttc aga gt<br>ggc tgc ttg gag caa aat ag

Primers are designed for 60°C annealing temperature. m = mouse
